# Longitudinal changes in MEG-based brain network topology of ALS patients with cognitive/behavioral impairment—An exploratory study

**DOI:** 10.1162/netn_a_00450

**Published:** 2025-07-17

**Authors:** Rosanne Govaarts, Elliz P. Scheijbeler, Emma Beeldman, Matteo Fraschini, Alessandra Griffa, Marjolein M. A. Engels, Anneke J. van der Kooi, Yolande A. L. Pijnenburg, Marianne de Visser, Cornelis J. Stam, Joost Raaphorst, Arjan Hillebrand

**Affiliations:** Amsterdam UMC location University of Amsterdam, Neurology, Amsterdam, The Netherlands; Amsterdam Neuroscience – Neurodegeneration, Amsterdam, The Netherlands; Leiden University Medical Center, C.J. Gorter MRI Center, Radiology, Leiden, The Netherlands; Amsterdam UMC location Vrije Universiteit Amsterdam, Clinical Neurophysiology & MEG Center, Amsterdam, The Netherlands; Amsterdam UMC location Vrije Universiteit Amsterdam, Alzheimer Center Amsterdam, Neurology, Amsterdam, The Netherlands; University of Cagliari, Electrical and Electronic Engineering, Cagliari, Italy; Department of Radiology and Medical Informatics, University of Geneva, Geneva, Switzerland; Institute of Bioengineering, Center for Neuroprosthetics, École Polytechnique Fédérale De Lausanne (EPFL), Lausanne, Switzerland; Leenaards Memory Center, Lausanne University Hospital and University of Lausanne, Lausanne, Switzerland; Amsterdam Neuroscience – Brain Imaging, Amsterdam, The Netherlands; Amsterdam Neuroscience – Systems & Network Neuroscience, Amsterdam, The Netherlands

**Keywords:** Amyotrophic lateral sclerosis, Behavioral variant frontotemporal dementia, Magnetoencephalography, Resting-state, Minimum spanning tree, Functional network topology

## Abstract

Amyotrophic lateral sclerosis (ALS) with only motor impairment (ALS-pure motor) and the behavioral variant of frontotemporal dementia (bvFTD) are hypothesized to represent extreme ends of a disease spectrum, which encompasses ALS with cognitive/behavioral impairment (ALSci/bi). In this longitudinal magnetoencephalography (MEG) study, we investigated changes in brain network topology of ALSci/bi over time as compared with ALS-pure motor and bvFTD patients. Resting-state MEG was recorded in ALS-pure motor (*n* = 9), ALSci/bi (*n* = 16), and bvFTD (*n* = 16) at baseline and 5-month follow-up, projected to source space. The corrected version of the amplitude envelope correlation was applied to compute frequency-band-specific functional connectivity between brain regions, from which the backbone of the functional networks was constructed using the minimum spanning tree (MST) approach. Reference MSTs were computed based on the functional connectivity matrices for ALS-pure motor and bvFTD, against which the networks of ALSci/bi were compared. We showed that, at baseline, networks in the theta band of ALSci/bi patients were more similar to ALS-pure motor than bvFTD. At follow-up, ALSci/bi patients’ beta-band network similarity had moved away from ALS-pure motor and resembled bvFTD. In conclusion, our findings suggest that brain networks of ALSci/bi patients move along the ALS-bvFTD spectrum over time, from ALS-pure motor to bvFTD-like topology.

## Author Summary

In this longitudinal magnetoencephalography (MEG) study, we explored changes in brain network topology in amyotrophic lateral sclerosis (ALS) patients with cognitive/behavioral impairment (ALSci/bi), compared with the two extreme ends of the ALS behavioral variant of frontotemporal dementia (ALS-bvFTD) spectrum (ALS with only motor impairment and bvFTD patients without ALS). We recorded resting-state MEG at baseline and 5-month follow-up, analyzing frequency-band-specific functional connectivity and constructing the networks’ backbone using the minimum spanning tree (MST). Our findings indicate that at baseline, ALSci/bi networks were more similar to ALS-pure motor than bvFTD in the theta band. At follow-up, ALSci/bi networks shifted toward bvFTD-like topology in the beta band. This suggests that ALSci/bi patients’ brain networks evolve along the ALS-bvFTD spectrum, highlighting potential implications for disease progression.

## INTRODUCTION

Amyotrophic lateral sclerosis (ALS) is a neurodegenerative disease that is primarily characterized by a relentlessly progressive loss of motor neurons, leading to a fatal outcome in 3–5 years (Hardiman et al., 2017). Approximately 30% of ALS patients also have nonmotor involvement ranging from mild to severe cognitive and/or behavioral impairment (ALSci/bi), and ALS with the concomitant behavioral variant of frontotemporal dementia (ALS-bvFTD) is found in 5%–10% (Burrell et al., 2016; Lillo et al., 2012; Strong et al., 2017; Trojsi et al., 2018).

There is debate as to whether a true disease spectrum exists between ALS and bvFTD, with, on one end, ALS with only motor impairment (ALS-pure motor) and, on the other end, bvFTD (without ALS). The position of ALS patients with mild cognitive and behavioral changes within this presumed spectrum is unclear. It is also unknown whether these patients have stable brain dysfunction over time or whether they exhibit progressive disturbances of their brain networks and, as such, move toward a bvFTD network phenotype (Bersano et al., 2020; Crockford et al., 2018).

Imaging may aid in providing objective measures of brain dysfunction in ALS. Magnetic resonance imaging (MRI) and positron emission tomography (PET) studies have shown that cognitive and/or behavioral impairment is related to the extent of structural and functional changes in nonmotor regions (Canosa et al., 2021; Cividini et al., 2022; Thome et al., 2022). Longitudinal structural MRI studies on ALS patients have shown progressive degeneration of white and gray matter in cortical (e.g., frontal and temporal regions) and subcortical regions (Benbrika et al., 2021; Ishaque et al., 2022; van der Burgh et al., 2020; van der Graaff et al., 2011; Westeneng et al., 2015). Longitudinal functional MRI studies have shown an association between cognitive/behavioral impairment and frontoparietal and default mode network connectivity (Agosta et al., 2013; Basaia et al., 2020; Castelnovo et al., 2020; Hu et al., 2020; Menke et al., 2017; Schulthess et al., 2016; Shen et al., 2018; Trojsi et al., 2021).

In addition to MRI, neurophysiological techniques (EEG and magnetoencephalography [MEG]) may provide an insight into abnormal brain networks in relation to impairment of cognition and behavior in ALS-pure motor, ALSci/bi, and bvFTD patients (Cosi et al., 1984; Dukic et al., 2019; McMackin et al., 2019, 2020, 2021; Polverino et al., 2022; Proudfoot, Bede, & Turner, 2018; Trojsi et al., 2018).

Some cross-sectional EEG and MEG studies in ALS have shown correlations between measures of network topology and cognitive impairment (Dukic et al., 2019), whereas others did not (Fraschini et al., 2016; Sorrentino et al., 2018). These studies included prevalent ALS patients (examined at least >1 year since diagnosis, with possibly slower disease progression) or did not extensively characterize their patients in terms of cognitive and/or behavioral impairment, complicating the interpretation of results. EEG and MEG have whole-brain coverage with high temporal resolution, yet MEG generally (i.e., when no extensive head modeling or high-density recordings are performed for EEG) has a higher spatial resolution and can detect subcortical activity (Baillet, 2017; Hillebrand, Nissen, et al., 2016; Pizzo et al., 2019). In a previous cross-sectional study, we showed that MEG in early symptomatic ALS patients reveals changes in resting-state functional connectivity in frontal, limbic, and subcortical regions that overlap considerably with bvFTD (Govaarts et al., 2022).

Longitudinal studies using MEG, which allows detailed investigation of global and regional brain (dys)function, may contribute to a better understanding of the disease process underlying the progressive motor and nonmotor neuron degeneration reported in ALS patients, especially within the ALS-bvFTD spectrum (Proudfoot, Colclough, et al., 2018; Sami et al., 2018).

In the current study, we report on longitudinal data from ALS-pure motor, ALSci/bi, and bvFTD patients. The aim of this study was twofold: First, we investigated to what extent resting-state functional networks of ALSci/bi patients resemble those of the extreme ends of the ALS-bvFTD spectrum (ALS-pure motor and bvFTD). Second, we examined if and how ALS patients move along the ALS-bvFTD spectrum over time. We hypothesized that resting-state brain networks in ALSci/bi patients at baseline would show similarities with those of both ends of the spectrum. We further expected these networks to change over time, where patterns of brain network abnormalities in ALSci/bi were expected to become more similar to those of patients with bvFTD. We used extensive neuropsychological examination, including disease-specific measures of cognition and behavior, and advanced MEG analysis methods, including reference minimum spanning trees (MST), to capture whether networks overlap (van Dellen et al., 2018). This allows us to quantify the overlap between brain networks of individual ALSci/bi patients and the extremes of the hypothesized ALS-bvFTD spectrum over time and to test these hypotheses.

## METHODS

### Participants

As previously described, ALS patients were prospectively recruited between September 2013 and December 2016 from a tertiary referral center in The Netherlands (Amsterdam University Medical Centres [UMC]; Beeldman et al., 2020). Patients with a symptom duration of less than 12 months were included. Cases could be sporadic or familial and had to have an upright forced vital capacity (FVC) > 70% of the predicted value (Beeldman et al., 2020). Patients were diagnosed with probable, laboratory-supported, or definite ALS, as defined by the El Escorial criteria (Brooks et al., 2000). They could have concomitant bvFTD (either possible or probable bvFTD according to Rascovsky criteria; Rascovsky et al., 2011).

BvFTD patients (long-standing without concomitant ALS; positive control group) were diagnosed with possible or probable bvFTD at a tertiary referral center for dementia (The Amsterdam Dementia Cohort; Rascovsky et al., 2011; van der Flier et al., 2014). They could be sporadic or familial cases and were included irrespective of disease duration or genotype (Beeldman et al., 2020).

The local medical ethical committee of the Amsterdam UMC approved the study, which was performed in agreement with the Declaration of Helsinki. Written informed consent was obtained from all participants at inclusion.

### Assessment of Cognition and Behavior

In order to be able to make a distinction between ALS patients with and without cognitive and behavioral impairment, all participants underwent a comprehensive neuropsychological examination and their proxy filled in the ALS-FTD-Questionnaire (ALS-FTD-Q) at baseline and 5-month follow-up (Beeldman et al., 2020; Raaphorst et al., 2012).

The neuropsychological examination comprised tests of executive function, language, memory, visuospatial functions, and social cognition. Test scores below the fifth percentile were considered abnormal, corrected for age, gender, and education, according to consensus criteria for cognitive impairment in ALS (Beeldman et al., 2020; Strong et al., 2017). The ALS-FTD-Q is a validated disease-specific proxy-based questionnaire for the screening of behavioral impairment in ALS (Raaphorst et al., 2012).

ALS patients without cognitive and behavioral impairment at baseline and follow-up were classified as ALS-pure motor and ALS patients with cognitive and/or behavioral impairment, at either baseline or follow-up, as ALSci/bi (including ALS-bvFTD patients; Beeldman et al., 2020; Raaphorst et al., 2012; Strong et al., 2017).

### MEG Data Analyses

MEG data acquisition and preprocessing were the same as described by Govaarts et al. (2022).

MEG recordings were performed at baseline and 5-month follow-up, prior to an MRI brain scan. All MEG recordings were performed with the participants in supine position in a magnetically shielded room (Vacuumschmelze GmbH, Hanau, Germany) using a 306-channel whole-head Vectorview system (Elekta Neuromag Oy, Helsinki, Finland). The recording protocol consisted of 5 min of eyes-closed task-free resting-state condition followed by a 2-min eyes-open and, again, a 5-min eyes-closed recording. Only the first eyes-closed recording was used for further analyses. The recordings were sampled at 1,250 Hz, with an online anti-aliasing (410 Hz) and high-pass (0.1 Hz) filter. After visual inspection of the neurophysiological signals (NS and RG) and after applying the extended cross-validated Signal Space Separation (SSS) method (van Klink et al., 2017), between 1 and 12 malfunctioning channels were excluded. Artifacts were subsequently removed with the temporal extension of SSS (Taulu & Hari, 2009; Taulu & Simola, 2006), using a subspace correlation limit of 0.9 and a window length of 10 s.

For anatomical reference, a high-resolution 3D T1 MRI scan was obtained (1-mm^3^ resolution; 3.0-T Ingenia scanner with a 32-channel receive-only head coil). The co-registration between MEG and MRI was performed using a surface-matching approach, and the co-registration was visually inspected for all participants. The co-registered MRI was spatially normalized to a template MRI (Hillebrand et al., 2012). The Automated Anatomical Labelling (AAL) atlas was subsequently used to label the voxels in 78 cortical and 12 subcortical regions of interest (ROIs; Tzourio-Mazoyer et al., 2002) and was transformed back to native space (Gong et al., 2009; Tzourio-Mazoyer et al., 2002). Each ROI’s centroid was used as a representative for that particular ROI (Hillebrand, Tewarie, et al., 2016). Beamforming was used to project broadband (0.5–48 Hz) filtered sensor signals to these centroid voxels (Hillebrand, Tewarie, et al., 2016). For computation of the beamformer weights (Hillebrand & Barnes, 2005; Hillebrand et al., 2005), the sphere that best fitted the scalp surface obtained from the co-registered MRI was used as a volume conductor model, together with an equivalent current dipole model (with optimum orientation determined using singular value decomposition; Sekihara et al., 2004) and the broadband data covariance matrix, which was based on 265 s of data on average (range: 127–375). Projection of the sensor-level MEG data through the normalized beamformer weights (Cheyne et al., 2007) resulted in broadband time series for each centroid of the 90 ROIs. For each subject, eight nonoverlapping artifact-free epochs, each with a length of 16,384 samples (13.1072 s), were selected from these time series, based on semiautomatic analysis and visual inspection (see Govaarts et al., 2022 for details and Supporting Information “Epoch selection”).

### Functional Connectivity

Functional connectivity between pairs of regions was computed for each epoch separately and subsequently averaged over epochs. As reported previously, we used the corrected version of the amplitude envelope correlation (AECc) as a metric of functional connectivity, for which the eight epochs were downsampled by a factor 4, and the AECc was computed for the following frequency bands: delta (0.5–4 Hz), theta (4–8 Hz), alpha1 (8–10 Hz), alpha2 (10–13 Hz), beta (13–30 Hz), and gamma (30–48 Hz; Govaarts et al., 2022). The alpha band was divided into an upper and lower band as these bands are involved in different cognitive processes (Klimesch et al., 1999).

### MST

The AECc-based connectivity matrices were used to construct frequency-band-specific MSTs. To construct the MST, we used Kruskal’s algorithm (Kruskal, 1956), which ranks pairwise connections from the lowest to highest weight. It starts with *N* disconnected nodes and adds the link with the lowest weight between two nodes, forming the smallest possible tree. Next, the second lowest weight link is added, either connecting two new nodes or linking to the current tree. This is repeated until all nodes in the network are connected. If adding a new link would result in a cycle or loop, the connection is discarded, and the next link ranked by weight is added. Since we are interested in the strongest connections of the network, inverted AECc values (1/AECc value) were used as input for the algorithm. Formally, we have therefore reconstructed *maximum* spanning trees.

An MST is a binary connected subgraph (i.e., edges exist or do not exist, they do not have weights) that captures the backbone of the underlying network. When constructed from a connected weighted graph with unique weights, the subgraph is unique. This allows for direct comparison of MSTs of different weighted graphs with the same number of nodes and without the need for arbitrary thresholding of the networks (van Wijk et al., 2010).

To identify the position of individual ALSci/bi patients along the ALS-bvFTD spectrum, we first constructed two reference MSTs (MST_ref_): MST_ref_-motor and MST_ref_-bvFTD. Each MST_ref_ was based on the average connectivity matrix of all subjects in the corresponding diagnostic group (ALS-pure motor and bvFTD). Next, MSTs were constructed for each individual ALSci/bi patient. MSTs were computed for each epoch (*n* = 8) separately and subsequently averaged over epochs.

**Figure 1.**
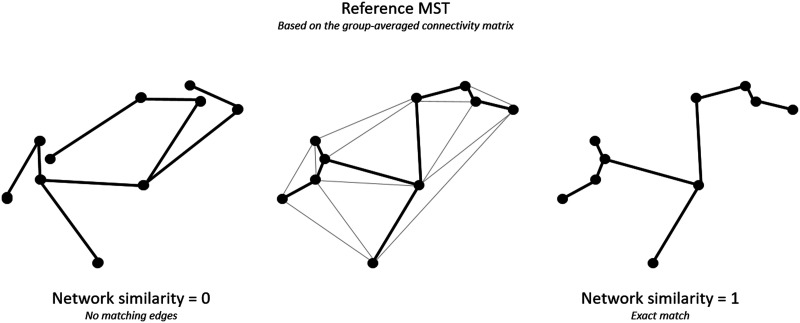
Quantifying network similarity. After an MST_ref_ has been computed from the ALS-pure motor or bvFTD group-averaged connectivity matrix (middle panel; light gray lines indicate connections that were present in the full weighted network, but that were not included in the MST), similarity subject-specific MSTs can be quantified by computing the fraction of edges that is present in both subgraphs. This ranges from 0 (no matching edges, left panel) to 1 (exact match, right panel).

The network similarity between the MST of individual ALSci/bi subjects (MST_individual_) and each MST_ref_ was quantified by calculating the fraction of edges that were present in both the MST_individual_ and MST_ref_ for the single epochs and taking the average over the eight epochs per subject. *Network similarity* ranged between 0 (no matching edges) and 1 (exact match; Figure 1; Boersma et al., 2013; Tewarie et al., 2015; van Dellen et al., 2015). At baseline and follow-up, the overlap of ALSci/bi patients was compared with MST_ref_-motor and MST_ref_-bvFTD was computed based on the network similarity. In addition, the longitudinal changes of the overall network organization were evaluated by comparing network similarity between baseline and follow-up.

When group differences were found for network similarity, these differences were further characterized with measures of network topology. Based on the MST, tree hierarchy, leaf fraction, and diameter were calculated for global analysis. To determine if the centrality of specific regions differed, the betweenness centrality was calculated for each region (see Supporting Information for details).

### Statistical Analysis

Demographic data and disease variables were summarized as mean and standard deviation (*SD*), median and range, or number and percentage, as appropriate. Group differences between ALS-pure motor, ALSci/bi, and bvFTD patients were analyzed using Kruskal–Wallis or chi-square tests, followed by post hoc Mann–Whitney *U* tests. At baseline and follow-up, Wilcoxon signed-rank tests were used to compare the overlap between ALSci/bi and ALS-pure motor with the overlap between ALSci/bi and bvFTD in all six frequency bands. Longitudinally, Wilcoxon signed-rank tests were used to evaluate changes in network topology between ALSci/bi and ALS-pure motor at baseline and follow-up and to evaluate changes in network topology between ALSci/bi and bvFTD at baseline and follow-up. False discovery rate (FDR) was applied to correct for multiple comparisons (six frequency bands and two groups; Benjamini & Hochberg, 1995). Statistical analyses were performed in Statistical Package for the Social Sciences, version 28. A two-sided *p* value below 0.05 was considered statistically significant.

## RESULTS

### Participants

Nine ALS-pure motor, 16 ALSci/bi patients, and 16 bvFTD patients were included. Follow-up data were obtained at a mean of 5.4 months (*SD* = 0.8). Characteristics of the patients at baseline are summarized in Table 1. A group effect was found for education (*H*(2) = 7.018, *p* = 0.030), disease duration (*H*(2) = 28.76, *p* < 0.001), Hospital Anxiety Depression Scale (HADS) – depression (*H*(2) = 7.17, *p* = 0.028), and ALS-FTD-Q (*H*(2) = 39.437, *p* = <0.001). Post hoc analysis showed an effect in disease duration between ALS-pure motor and bvFTD (*U* = 0, *Z* = −4.08, *p* < 0.001) and between ALSci/bi and bvFTD (*U* = 0, *Z* = −4.83, *p* < 0.001). HADS – depression score differed between ALS-pure motor and bvFTD (*U* = 26, *Z* = −2.68, *p* = 0.007), and education differed between ALSci/bi and bvFTD (*U* = 57.5, *Z* = −2.52, *p* = 0.012). ALS-FTD-Q differed between ALS-pure motor and ALSci/bi (*U* = 16.5, *Z* = −3.148, *p* = 0.002), ALS-pure motor and bvFTD (*U* = 0, *Z* = −4.079, *p* < 0.001), and between ALSci/bi and bvFTD (*U* = 20.5, *Z* = −4.054, *p* < 0.001).

**Table 1.** Participant characteristics and disease variables at baseline

	ALS-pure motor *n* = 9	ALSci/bi *n* = 16	bvFTD *n* = 16	*p* value
Age (years)	64.9 (8.7)	61.0 (7.1)	65.6 (8.0)	0.709
Male (*n*, %)	4 (44)	12 (75)	12 (75)	0.220
Education (years)	14 (12–18)	12 (8–18)	14 (10–18)	**0.030** ^
Disease duration (months)	10 (7–14)	11 (6–15)	33 (18–168)	**<0.001** * ^
Site of onset (l/b/lb)	5/3/1	7/8/1	n/r	0.704
ALSFRS-R score	43 (37–45)	42 (31–46)	n/r	0.647
ALSFRS-R slope	0.50 (0.38–1.22)	0.67 (0.30–2.43)	n/r	0.713
FVC (%pred)	101 (87–142)	91 (67–116)	n/r	0.089
*C9orf72* mutation^†^ (*n*, %)	1 (11)	1 (6)	3 (19)	0.312
Riluzole^††^ (*n*, %)	9 (100)	16 (100)	n/r	–
HADS - anxiety	3 (0–11)	5 (0–11)	5.5 (0–9)	0.394
HADS - depression	0 (0–3)	1 (0–10)	2.5 (0–8)	**0.028** ^#^
ALS-FTD-Q	4 (0–10)	14.5 (3–46)	46.5 (13–63)	**<0.001** * ^#^ ^

Data are expressed as mean (*SD*) or median (range), where appropriate. A group effect was found for education (*H*(2) = 7.018, *p* = 0.030), disease duration (*H*(2) = 28.76, *p* < 0.001), HADS – depression (*H*(2) = 7.17, *p* = 0.028), and ALS-FTD-Q (*H*(2) = 39.437, *p* < 0.001). Post hoc analysis showed an effect in disease duration between ALS-pure motor and bvFTD (*U* = 0, *Z* = −4.08, *p* < 0.001) and between ALSci/bi and bvFTD (*U* = 0, *Z* = −4.83, *p* < 0.001). HADS – depression score differed between ALS-pure motor and bvFTD (*U* = 26, *Z* = −2.68, *p* = 0.007), and education differed between ALSci/bi and bvFTD (*U* = 57.5, *Z* = −2.52, *p* = 0.012). There was no difference as regards to education between ALS-pure motor and ALSci/bi patients. ALS-FTD-Q differed between ALS-pure motor and ALSci/bi (*U* = 16.5, *Z* = −3.148, *p* = 0.002), ALS-pure motor and bvFTD (*U* = 0, *Z* = −4.079, *p* < 0.001), and between ALSci/bi and bvFTD (*U* = 20.5, *Z* = −4.054, *p* < 0.001). ci/bi: cognitive and/or behavioral impairment, including ALS-bvFTD; *n*: number; site of onset (l/b/lb): limb onset/bulbar onset/limb and bulbar onset; ALSFRS-R: ALS Functional Rating Scale–Revised, a score of 48 indicates no physical impairment; ALSFRS-R slope: calculated as 48 − ALSFRS-R score at time of MEG scan divided by the number of months between first symptoms and MEG scan; FVC %pred: FVC in upright position, percentage of predicted value; HADS: Hospital Anxiety Depression Scale, a higher score indicates more anxiety/depression, with a clinical threshold of 7 on the subscales for anxiety and depression; ALS-FTD-Q: ALS-FTD questionnaire; n/r = not relevant.

^†^ *C9orf72* mutation status was the only mutations that was tested and was missing in three ALSci/bi patients and six bvFTD patients.

^††^ Median treatment duration was 12 (5–39) weeks; one ALS-pure motor patient was treated for 5 weeks, and one ALSci/bi patient was treated for 7 weeks; all other patients were treated for at least 8 weeks.

* Difference between ALS-pure motor and ALSci/bi (*p* < 0.05).

^#^ Difference between ALS-pure motor and bvFTD (*p* < 0.05).

^ Difference between ALSci/bi and bvFTD (*p* < 0.05).

### MST—Network Similarity

**Figure 2.**
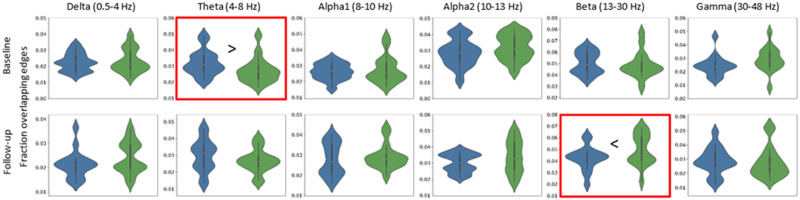
ALSci/bi similarity to reference networks for ALS-pure motor and bvFTD. Violin plots of network similarity between ALSci/bi versus ALS-pure motor (blue) and ALSci/bi versus bvFTD (green). Red boxes indicate significant differences in network similarity between ALSci/bi versus ALS-pure motor (theta [*M* ± *SD*] 0.032 ± 0.008; beta 0.042 ± 0.010) and ALSci/bi versus bvFTD (theta 0.027 ± 008; beta 0.049 ± 0.013) at a single time-point. bvFTD: behavioral variant frontotemporal dementia; ci/bi: cognitive and/or behavioral impairment, including ALS-bvFTD. Values are based on the mean over epochs (*n* = 8) of every patient. See also Supporting Information Table S1 for the raw data, expressed as mean (*SD*).

At baseline, ALSci/bi patients showed more overlap (i.e., higher network similarity) with the MST_ref_-motor (network similarity = 0.032 ± 0.008) than the MST_ref_-bvFTD (network similarity = 0.027 ± 0.008) in the theta band, which remained significant after FDR correction (*Z* = −2.50, *p* = 0.012; Figure 2, Supporting Information Table S1). ALSci/bi patients at follow-up showed higher network similarity to MST_ref_-bvFTD (network similarity = 0.049 ± 0.013) than MST_ref_-motor (network similarity = 0.042 ± 0.010) in the beta band, which remained significant after FDR correction (*Z* = −2.38, *p* = 0.017; Figure 2, Supporting Information Table S1), while no significant differences were found for the other frequency bands, including the theta band at follow-up (Supporting Information Figures S1–S4).

We compared the similarity of the ALSci/bi group to each MST_ref_ between baseline and follow-up. After FDR correction, no significant differences in network similarity were found between baseline and follow-up.

**Figure 3.**
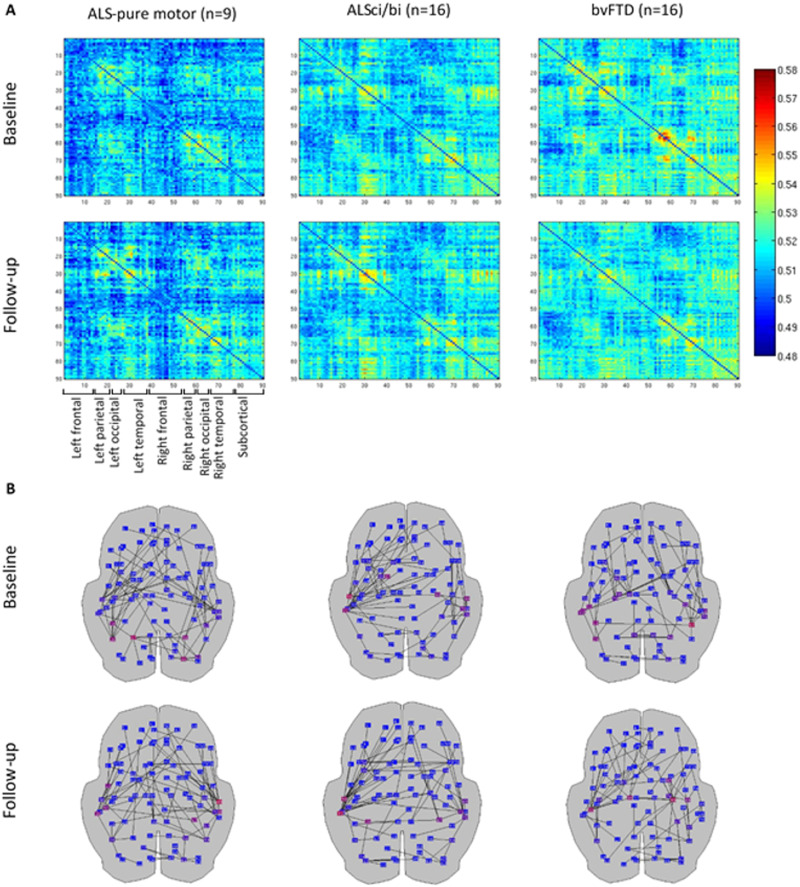
Illustrative overview of theta band network similarity at baseline and follow-up. (A) Theta band functional connectivity matrices weighted by the corrected version of the AECc averaged across all epochs and subjects within each group. The first row represents the baseline measurement; the second row represents the follow-up measurement. Each column shows results for the AECc, for ALS-pure motor, ALSci/bi, and bvFTD. The same color scale was used for both time points and all groups. The ROIs are obtained from the AAL atlas. (B) Theta band MSTs based on the average adjacency matrices (in A) are displayed on a schematic of the brain (axial view). The first row represents the baseline measurement; the second row represents the follow-up measurement. Columns show MSTs for ALS-pure motor, ALSci/bi, and bvFTD. Red, purple, and blue dots indicate ROIs from high, to medium, and to low node betweenness centrality, respectively.

**Figure 4.**
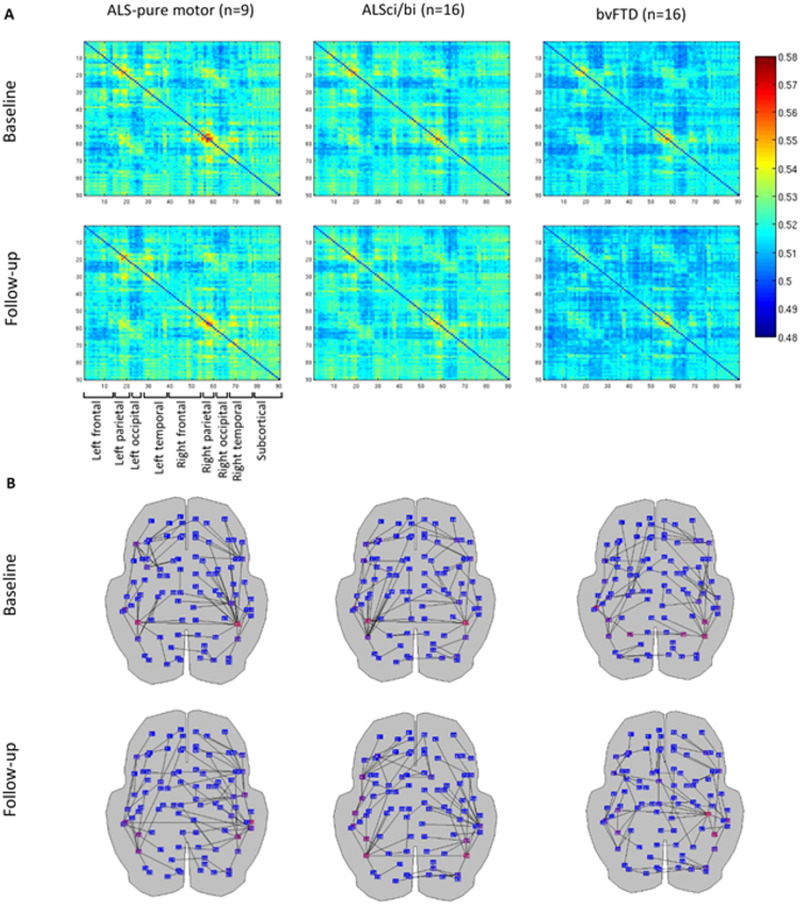
Illustrative overview of beta band network similarity at baseline and follow-up. (A) Beta band functional connectivity matrices weighted by the corrected version of the AECc averaged across all epochs and subjects within each group. The first row represents the baseline measurement; the second row represents the follow-up measurement. Each column shows results for the AECc, for ALS-pure motor, ALSci/bi, and bvFTD. The same color scale was used for both time points and all groups. The ROIs are obtained from the AAL atlas. (B) Beta band MSTs based on the average adjacency matrices (in Figure 3A) are displayed on a schematic of the brain (axial view). The first row represents the baseline measurement; the second row represents the follow-up measurement. Columns show MSTs for ALS-pure motor, ALSci/bi, and bvFTD. Red, purple, and blue dots indicate ROIs from high, to medium, and to low node betweenness centrality, respectively.

To illustrate the underlying data of the results, the group-averaged connectivity matrices (Figures 3A and 4A and Supporting Information Figures S1 and S2), as well as the MSTs based on these group-averaged matrices (Figures 3B and 4B and Supporting Information Figures S3 and S4), are visualized for the theta (Figure 3) and beta band (Figure 4). The connectivity matrices reveal the strength of the connections between the individual regions, with strong connections in dark red/blue colors.

### MST Characteristics

Several characteristics of the MST of each ALSci/bi patient and of the MST_ref_ groups were calculated to further quantify network topology (i.e., tree hierarchy, diameter, leaf fraction [global analysis] and betweenness centrality [regional analysis]). No significant differences were found between groups or within group between baseline and follow-up (see Supporting Information).

## DISCUSSION

By applying MST analysis to source space MEG data, we were able to compare the brain network topology of ALS-pure motor, ALSci/bi, and bvFTD patients. We aimed (a) to identify the position of ALSci/bi patients on the presumed ALS-bvFTD spectrum at baseline and (b) to map movement along this spectrum over a 5.4-month (*SD* = 0.8) period. We hypothesized that the baseline network topology of ALSci/bi patients would show similarities with both groups, with a shift toward more bvFTD-like topology with the progression of the disease. The results indicated that at baseline, brain networks of ALSci/bi patients showed more similarity to the reference network of the ALS-pure motor than that of the bvFTD group in the theta band. However, after a 5.4-month (*SD* = 0.8) period, the network topology of ALSci/bi patients appears to shift toward the bvFTD group. This is reflected by the higher similarity to MST_ref_-bvFTD than MST_ref_-pure motor at follow-up in the beta band. No significant differences were reported regarding MST characteristics (i.e., three hierarchy, leaf fraction, diameter, and betweenness centrality).

This study included a well-characterized longitudinal cohort of ALS-pure motor, ALSci/bi, and bvFTD patients. Previous studies on cognitive and behavioral impairment in ALS mostly did not include bvFTD patients, hampering the investigation of the ALS-bvFTD spectrum in full.

EEG and MEG studies—often cross-sectional—with larger sample sizes (up to *n* = 100) have revealed changes in oscillatory power (e.g., decreased parieto-occipital alpha power) and functional connectivity (e.g., increased frontoparietal alpha and beta connectivity) in ALS patients compared with healthy controls (Dukic et al., 2019; Fraschini et al., 2016; Iyer et al., 2015; Jayaram et al., 2015; Mai et al., 1998; Nasseroleslami et al., 2019; Polverino et al., 2022; Proudfoot, Colclough, et al., 2018; Santhosh et al., 2005; Trubshaw et al., 2024). However, findings on abnormalities in the topology of functional networks in ALS have been less consistent, and so far, only one study showed a correlation between cognition and network disruption (Dukic et al., 2019).

Fraschini and colleagues reported that the MSTs derived from EEG data of ALS patients had a significantly lower leaf fraction in the beta band compared with healthy controls. Additionally, leaf fraction showed a negative correlation with motor disability in ALS. No significant differences were reported for tree hierarchy or diameter (Fraschini et al., 2016). In a MEG study, Sorrentino et al. (2018) showed no significant differences between early-stage ALS patients (King’s Stages 1 and 2 and healthy controls), while advanced ALS patients (King’s Stages 3 and 4) did show modified brain topology in the delta, theta, beta, and gamma frequency bands compared with healthy controls. Brain networks became more centralized as ALS patients progressed to later stages of the disease (Sorrentino et al., 2018).

A MEG study on the flexibility of brain dynamics showed a correlation with disease stage and symptom severity, within theta and delta bands (Polverino et al., 2022). In addition, this study showed that reduced flexibility could predict these clinical scores. A more recent MEG study showed reduced sensorimotor beta power and an association between increased motor disability and increased global connectivity in the theta band (Trubshaw et al., 2024). Instead of constructing the MST, Iyer and colleagues (2015) binarized the networks using an arbitrary threshold of 20% and reported that ALS patients without cognitive complaints have increased clustering coefficients in the alpha and gamma bands. Another EEG study used non-negative matrix factorization to reconstruct AECc-based networks, showing that theta band connectivity in the frontoparietal network correlated with cognitive functioning (based on the Edinburgh Cognitive and Behavioural ALS Screen; Abrahams et al., 2014), and gamma band connectivity in frontotemporal networks correlated with language function. In addition, beta band functional connectivity (imaginary coherence) in the sensorimotor network correlated with motor impairment (Dukic et al., 2019).

In summary, the stage of the disease and the extent of cognitive impairment appear to be related to the level of network disruption in ALS patients (Dukic et al., 2019). In addition, Polverino et al. (2022) predicted based on brain flexibility and clinical disability as well motor impairment as cognitive and behavioral impairment. However, not all studies could confirm these results and showed power, connectivity, and network changes in multiple frequency bands. The abovementioned studies used a cross-sectional design, which hampers firm conclusions about disease progression and changes in network organization over time. Furthermore, these studies included patients with a disease duration ranging from 18 months from disease onset to more than 5 years (for one study, the disease duration is unknown [Fraschini et al., 2016]; Dukic et al., 2019; Iyer et al., 2015; Sorrentino et al., 2018). In our study, we aimed to include a cohort of ALS patients without too much heterogeneity, compared with other studies, in terms of disease progression or stage of the disease; all of the patients were early in the disease course (median disease duration at inclusion 11 months [median (range) ALS Functional Rating Scale–Revised (ALSFRS-R) of 42 (31–46)] and at follow-up 17 months [median (range) ALSFRS-R of 38 (16–45)]. This early stage of the clinical disease may explain the lack of significant findings on MST characteristics (i.e., tree hierarchy, leaf fraction, diameter, and betweenness centrality). Topological metrics are specific measures that may be less sensitive to subtle changes and, therefore, less suitable for small sample sizes or changes that present early in the disease course.

Another possible explanation for the divergence of our results in comparison with those of previous studies could be that some of the latter may have suffered from methodological biases when comparing groups or conditions, by ignoring the effects of different network densities on measures of network topology (Fornito et al., 2013; van Wijk et al., 2010). To avoid biases due to differences in thresholding, edge density, or edge weights that may hamper a meaningful comparison between groups or time points when using weighted/unweighted graph analyses (van Wijk et al., 2010), we constructed and compared MSTs of the original weighted graphs (Jackson & Read, 2010a, 2010b; Stam et al., 2014; Tewarie et al., 2015). MST analysis is costly though, in the sense that the subgraph may discard some information about network topology compared with an analysis of the full network. However, we have previously shown that MST characteristics are equally sensitive to alterations in network topology as conventional graph theoretical measures, such as the characteristic path length and node degree (Tewarie et al., 2015). Similarly, information about hierarchical structure is also retained in the MST (Yu et al., 2015). Furthermore, we assumed here that the signals were stationary within epochs. Alternative approaches that take the dynamic nature of the signals into account (Liuzzi et al., 2019; Tewarie et al., 2019) could be explored in future studies.

In our previous cross-sectional study, we showed a higher baseline functional connectivity in the delta and gamma bands in ALS and bvFTD compared with healthy controls (Govaarts et al., 2022). However, in this study, we did not find a difference in network topology in these frequency bands, possibly showing that a difference in functional connectivity does not necessarily imply a difference in network topology as well, although this finding could also be explained by differences in the group comparisons (comparison with healthy controls vs. comparison between patient groups). We did find a higher similarity in MST brain networks in the theta band between ALS-pure motor and ALSci/bi compared with bvFTD and ALSci/bi at baseline and a decrease in beta band network similarity at follow-up between ALS-pure motor and ALSci/bi. We did show a lower beta power in bvFTD patients compared with healthy controls in our previous study, which is in line with our findings in network topology, where ALSci/bi patients at follow-up showed a higher similarity with bvFTD compared with ALS-pure motor.

### Limitations and Future Research

While our cohort is well characterized (inclusion of ALS patients early in the disease course, comprehensive neuropsychological examination [Beeldman et al., 2020] including a disease-specific behavioral questionnaire [Raaphorst et al., 2012], and the inclusion of bvFTD patients without ALS), the main limitation of this study is that the number of patients per group was small, especially for the ALS-pure motor sample (*n* = 9). In this study, we had two measurements with a follow-up time of 5.4 months (*SD* = 0.8). The patient groups differed in educational level and disease duration; however, due to the small sample size, we could not test whether these potential confounding factors explained the observed effects. Due to the inclusion criterium of a symptom duration of less than 12 months, it is likely that we included ALS patients with a relatively fast disease progression. Of these patients, ALS patients with mainly progression of motor impairment are expected to remain near the ALS-pure motor side of the spectrum, although this hypothesis was not tested here. However, an advantage of the relatively short symptom duration is that all patients included in this study were able to undergo both measurements, whereas other longitudinal studies in ALS showed losses-to-follow-up of up to 72% (Westeneng et al., 2015).

The inclusion of epochs was based on the maximum number of artifact-free epochs that were available for all patients (*n* = 8), based on the first 5 min of eyes-closed, task-free, resting-state condition. With eyes closed, there is a risk that subjects become drowsy; therefore, during the recordings, drowsiness was monitored, and an acoustic stimulus was given when signs of drowsiness appeared. Moreover, when the eyes are open, patients with bvFTD tend to look around, instead of focusing on a fixation point, which causes artifacts. Additionally, during the eyes-closed state, the posterior dominant (alpha) rhythm is much more pronounced. Since the alpha rhythm has been implicated in ALS and bvFTD in previous studies (Dukic et al., 2019; Iyer et al., 2015; Sorrentino et al., 2018), we chose to analyze the eyes-closed state.

In addition, AECc was the only mode of functional connectivity used, as it has been shown to be a reliable measure of functional connectivity (Colclough et al., 2016) that is also sensitive to disease effects (Briels et al., 2020; Schoonhoven et al., 2022). Given the complementary nature of phase-based and amplitude-based coupling (Hindriks & Tewarie, 2023), future work could examine differences along the disease spectrum for functional networks that are based on phase-based measures, such as the phase lag index (particularly in the theta band [Schoonhoven et al., 2022], although these are generally more noisy; Colclough et al., 2016). Furthermore, studies may need to consider alternative measures of central tendency as the non-Gaussianity of functional connectivity and similarity scores may have introduced some biases in our results.

In addition, our measure of network similarity was estimated by computing the overlap of the MST for single epochs with an MST_ref_ and then averaging over epochs. Given that the adjacency matrix for a single epoch may be noisy, this approach may have resulted in slightly noisy estimates of MST similarity, thereby masking group differences. We reanalyzed the data using the MST of the mean adjacency matrix (i.e., averaging the adjacency matrices and then computing the MST), which, we hypothesized, would give a more stable result and thereby higher MST values. Although the similarity scores were indeed higher with the new analysis, they remained rather low (Supporting Information Table S3). A possible explanation could be that the MST_ref_ is still noisy due to the low sample size, although previous work has shown that MSTs are relatively stable in the presence of noise (Tewarie et al., 2015).

In addition, a longer follow-up time with multiple measurements, including ALS patients in different stages of the disease and bvFTD patients with a shorter disease duration, and/or a varying degree of cognitive/behavioral impairment, could give more insight into the position and transition of ALSci/bi along the ALS-bvFTD spectrum in combination with the correlation of clinical variables and the influence of specific frequency bands. Furthermore, an advanced longitudinal design would give the possibility to test for the predictive power of similarity with clinical progression.

## CONCLUSION

In this longitudinal MEG-based network study, we aimed to identify that ALSci/bi patients fall within the ALS-bvFTD spectrum at two time points. Using an MST_ref_ approach, we showed that at baseline, brain networks in the theta band of ALSci/bi patients resemble those of ALS-pure motor more than those of bvFTD. At follow-up, brain networks of the ALSci/bi group move in the direction of bvFTD-like topology in the beta band. Investigations in larger groups, including patients with a longer disease duration, and a longer follow-up time are needed to confirm these results.

## ACKNOWLEDGMENTS

The authors would like to acknowledge all patients and their caregivers for participating. We would also like to thank Nico Akemann, Karin Plugge, Marieke Alting Siberg, and Marlous van den Hoek for the MEG acquisitions, as well as Ndedi Sijsma for the MEG acquisitions and visual epoch inspection. Furthermore, we would like to thank our colleagues from the UMC Utrecht for their help in retrieving the C9orf72 status. Several authors of this publication are members of the Netherlands Neuromuscular Center (NL-NMD) and the European Reference Network for rare neuromuscular diseases (ERN-EURO-NMD).

## SUPPORTING INFORMATION

Supporting information for this article is available at https://doi.org/10.1162/netn_a_00450.

## AUTHOR CONTRIBUTIONS

Rosanne Govaarts: Formal analysis; Resources; Validation; Visualization; Writing – original draft; Writing – review & editing. Elliz P. Scheijbeler: Visualization; Writing – review & editing. Emma Beeldman: Conceptualization; Methodology; Resources; Writing – review & editing. Matteo Fraschini: Formal analysis; Writing – review & editing. Alessandra Griffa: Formal analysis; Resources; Writing – review & editing. Marjolein M. A. Engels: Resources; Writing – review & editing. Anneke J. van der Kooi: Resources; Writing – review & editing. Yolande A. L. Pijnenburg: Resources; Writing – review & editing. Marianne de Visser: Conceptualization; Methodology; Resources; Supervision; Writing – review & editing. Cornelis J. Stam: Conceptualization; Methodology; Writing – review & editing. Joost Raaphorst: Conceptualization; Methodology; Resources; Supervision; Validation; Writing – review & editing. Arjan Hillebrand: Formal analysis; Methodology; Resources; Supervision; Validation; Writing – review & editing.

## FUNDING INFORMATION

Marianne de Visser, Stichting ALS Nederland (https://dx.doi.org/10.13039/501100014076), Award ID: 2013-19. Rosanne Govaarts, Nederlandse Organisatie voor Wetenschappelijk Onderzoek (https://dx.doi.org/10.13039/501100003246), Award ID: 023.012.055.

## DATA AVAILABILITY STATEMENT

Data are available upon reasonable request.

## ETHICS APPROVAL

The local medical ethical committee of the Amsterdam UMC approved the study, which was performed in agreement with the Declaration of Helsinki.

## INFORMED CONSENT

Written informed consent was obtained from all participants.

## Supplementary Material



## TECHNICAL TERMS

Minimum spanning tree (MST): Connects all nodes in a weighted graph with the least total edge weight, avoiding cycles. Here, nodes represent brain regions and edges represent connections between them.
Cross-validated Signal Space Separation method (xSSS): Combining a physical magnetic field model (as in SSS) with a cross-validation approach to remove sensor noise and external noise enhances data clarity and accuracy.
Signal Space Separation (SSS): Uses a physical magnetic field model to distinguish between brain activity and external noise sources, resulting in clearer MEG data.
Temporal extension of Signal Space Separation method (tSSS): Enhancement of the Signal Space Separation method used to remove interference from noise sources that are (typically) close to the head.
Beamforming: Spatial filtering technique to estimate the time series of neuronal activation at specific locations in the brain.
Artifact-free epochs: Segments of MEG data that have been carefully selected to exclude any artifacts or noise.
Tree hierarchy: Characterizes the organization of brain networks that have a tree-like structure, quantifying the trade-off between large-scale integration and overload of central nodes (also called hubs).
Leaf fraction: This metric indicates the proportion of nodes in the network that have only one connection.
Diameter: Measures the longest shortest path between any two nodes in the network.
Betweenness centrality: The number of times a node acts as a bridge along the shortest path between two other nodes.
