# Supplementary material for: Longitudinal changes in MEG-based brain network topology of ALS patients with cognitive/behavioral impairment—An exploratory study
Source: Netw Neurosci. 2025 Jul 17;9(3):824–41. doi: 10.1162/netn_a_00450 (PMC12283152; doi:10.1162/netn_a_00450)
Supplement: Supplementary file 1 [file netn-9-3-824-s001.pdf]

Supplementary Material

Epoch selection

For each subject, eight non-overlapping artefact-free epochs, each with a length of 16,384 samples (13.1072 s), were selected out of the first 5 min eyes-closed recording. Eyes-open recordings were not analysed due to artefacts and reduced power of the alpha rhythm.

Epoch selection was based on the following criteria using a strict semi-automatic procedure implemented in Matlab, version 2018b: 1) discard epochs with extreme values in the temporal domain. Epochs where the signal amplitude of one or more channels exceeds the average range could be corrupted by artefacts. Typical artefacts were due to (eye) movements, swallowing or dental prosthetics; 2) discard epochs with individual alpha frequency values outside the range mean ± 2 standard deviation (SD) (mean and SD values computed over all the epochs of a single subject), as these could be indicative of drowsiness (Hari and Puce, 2017); and 3) select the ten epochs out of the remaining epochs with the highest individual alpha frequency and relative alpha1 power in occipital channels for that subject, to avoid possible drowsiness biases across subjects. Two examiners (RG and NS) independently evaluated the ten selected epochs: if the epochs (still) contained artefacts then they were excluded. This procedure allowed to selection an approximatively equivalent amount of data for each subject in an objective way and led to at least eight non-overlapping artefact-free epochs for each subject.

Table S1. ALSci/bi network similarity

	Similarity to MST_{ref}-motor		Similarity to MST_{ref}-bvFTD		BL vs BL	BL vs FU MST_{ref}-motor	BL vs FU MST_{ref}-bvFTD	FU vs FU
	Baseline	Follow-up	Baseline	Follow-up				
Delta	.021 (.006)	.021 (.006)	.023 (.007)	.024 (.007)	.623	.244	.737	.167
Theta	.032 (.008)	.030 (.010)	.027 (.008)	.027 (.007)	**.012**	.338	.918	.215
Alpha1	.026 (.005)	.028 (.008)	.028 (.009)	.029 (.006)	.649	.266	.453	.535
Alpha2	.029 (.009)	.030 (.006)	.032 (.008)	.033 (.010)	.163	.513	.408	.224
Beta	.050 (.010)	.042 (.010)	.049 (.012)	.049 (.013)	.423	.046*	.776	**.017**
Gamma	.024 (.008)	.029 (.009)	.029 (.009)	.028 (.025)	.127	.222	.211	.717

Note: Data are expressed as mean (SD) and based on the mean over epochs (n=8) for every patient. MST_{ref}: reference minimum spanning tree; motor: amyotrophic lateral sclerosis patients with only motor impairment; bvFTD: behavioural variant frontotemporal dementia; vs: versus; BL: Baseline; FU: follow-up . Bold: significant difference based on the Wilcoxon signed rank test, FDR corrected. *significant difference based on the Wilcoxon signed rank test, did not remain significant after FDR correction.

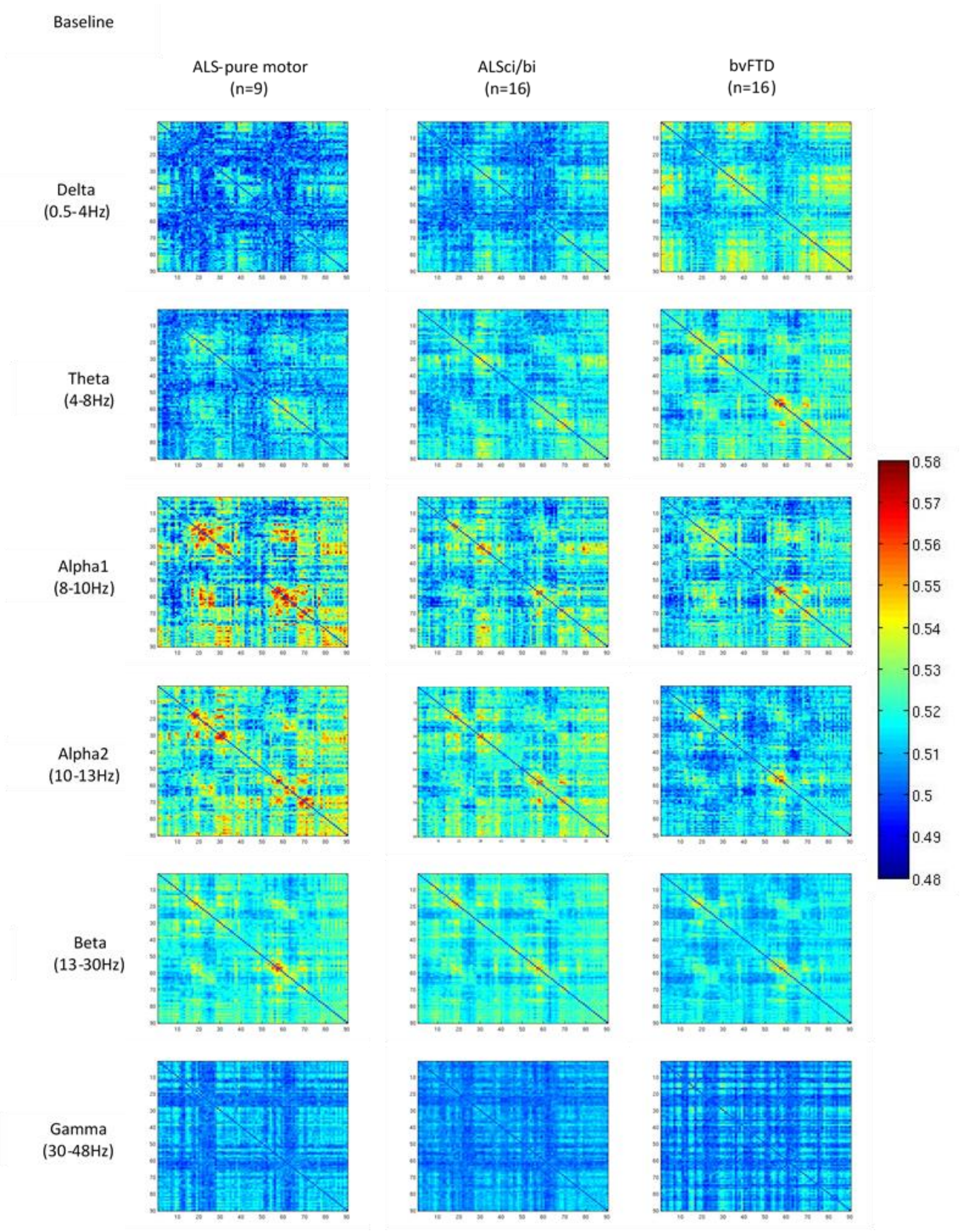

Figure S1. Baseline connectivity matrices of the corrected version of the amplitude envelope correlation (AECc)

Adjacency matrices of the corrected version of the amplitude envelope correlation (AECc) averaged across all epochs and subjects. Each column shows results for the AECc, for amyotrophic lateral sclerosis (ALS) patients – pure motor, ALS with cognitive and/or behavioural impairment including ALS-bvFTD (ALSci/bi), and behavioural variant frontotemporal dementia (bvFTD) patients. All measurements and groups show the matrices using the same colour scale. The ROIs are obtained from the AAL atlas. The matrices are ordered from left to right hemisphere in the following way: rows/columns 1-15 represent left frontal regions, 16-21 left parietal regions, 22-27 left occipital regions, 28-39 left temporal regions, 40-54 right frontal regions, 55-60 right parietal regions, 61-66 right occipital regions, 67-78 right temporal regions, and 79-90 subcortical regions.

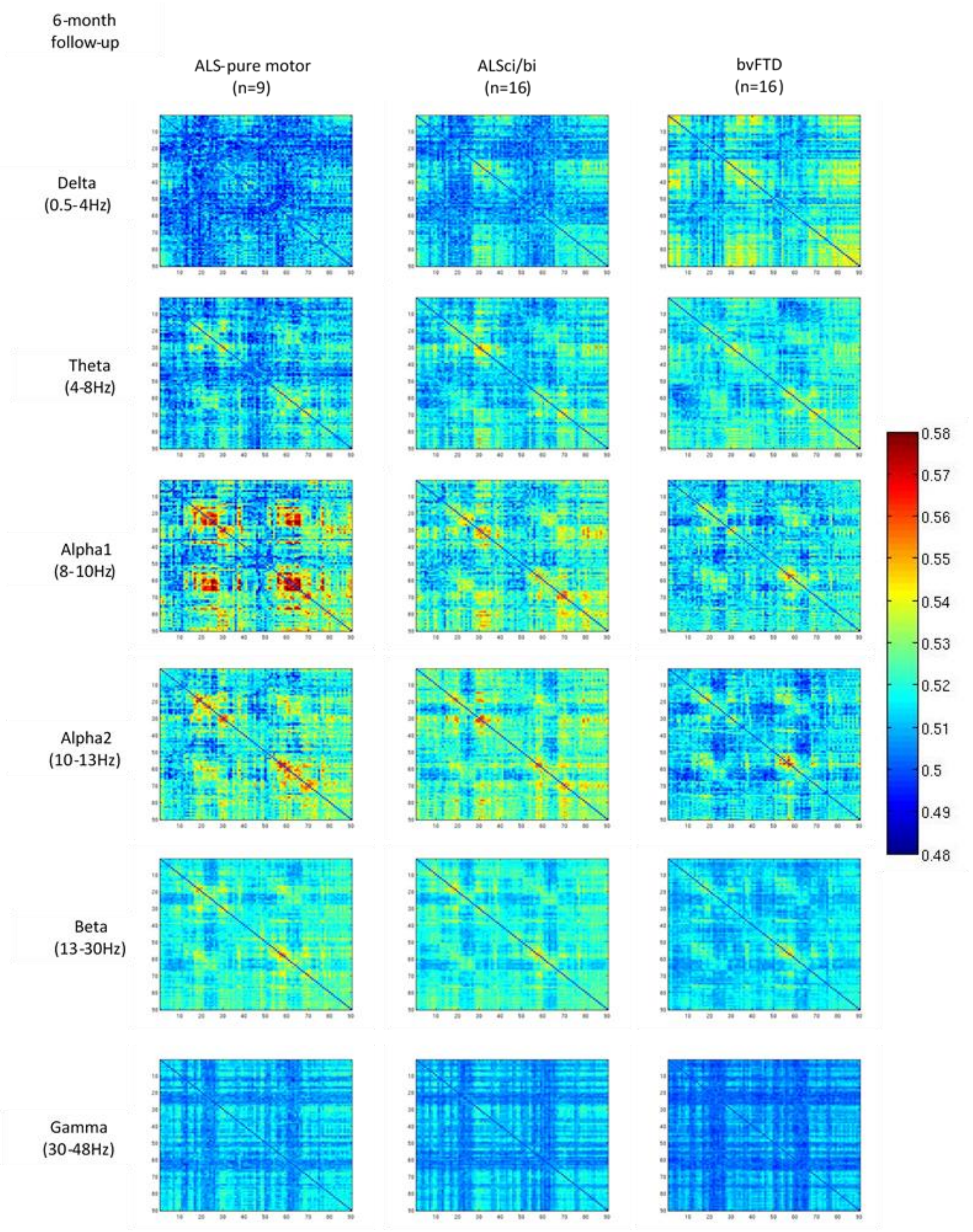

Figure S2. Follow-up connectivity matrices of the corrected version of the amplitude envelope correlation (AECc)

As in Figure S1, but for the follow-up recordings.

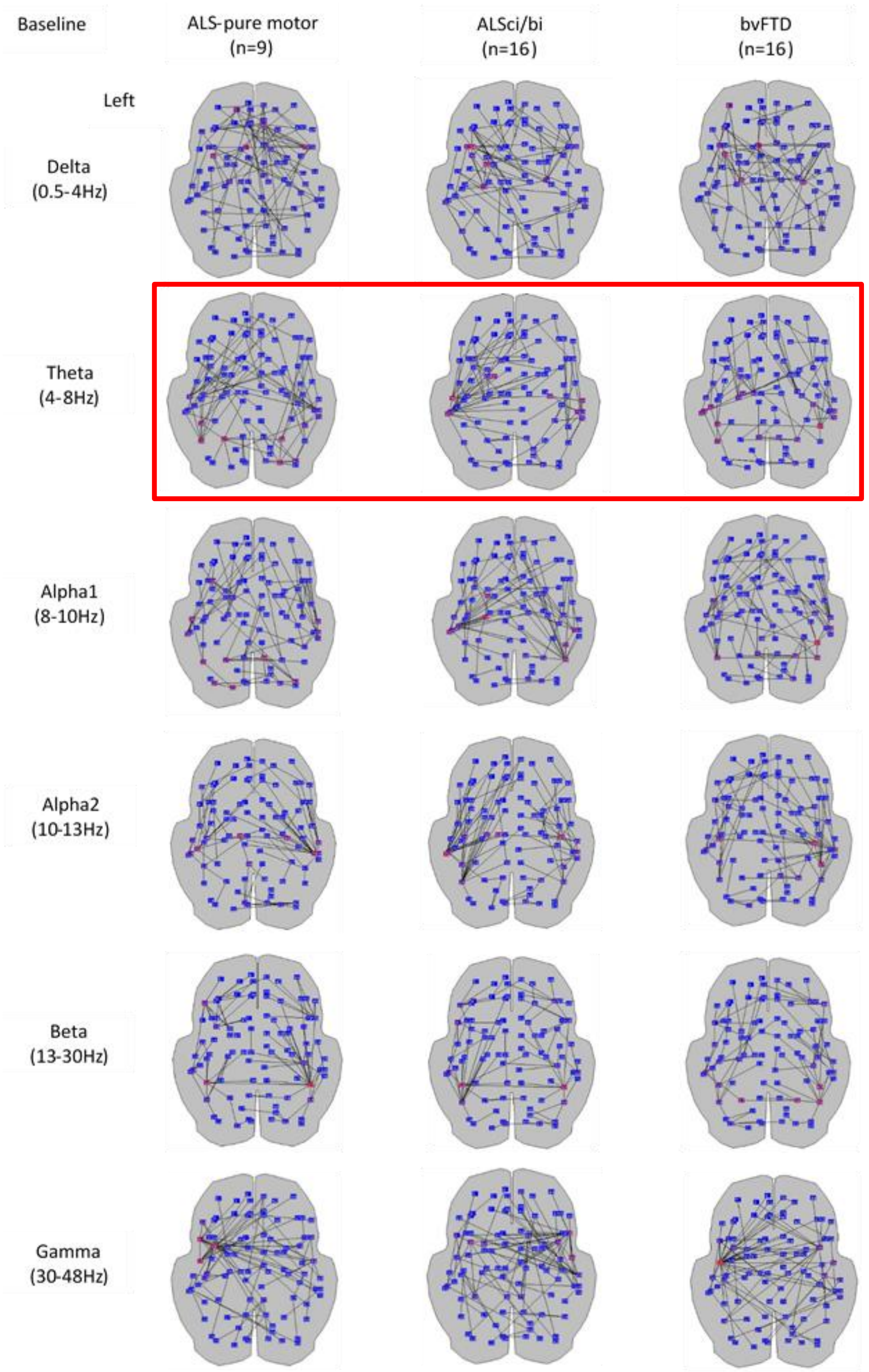

Figure S3. Baseline MST network changes in ALS-pure motor, ALSci/bi and bvFTD
MSTs based on the average adjacency matrices are displayed on a schematic of the brain (axial view). Each column shows results for the MST, amyotrophic lateral sclerosis (ALS) patients – pure motor, ALS with cognitive and/or behavioural impairment including ALS-bvFTD (ALSci/bi), and behavioural variant frontotemporal dementia (bvFTD) patients. Red, purple and blue dots indicate ROIs respectively from high, to medium, to low node betweenness. The red box indicates significant differences based on the Wilcoxon-signed tank test. Note: values for statistics are based on the mean over epochs (n=8) for every patient.

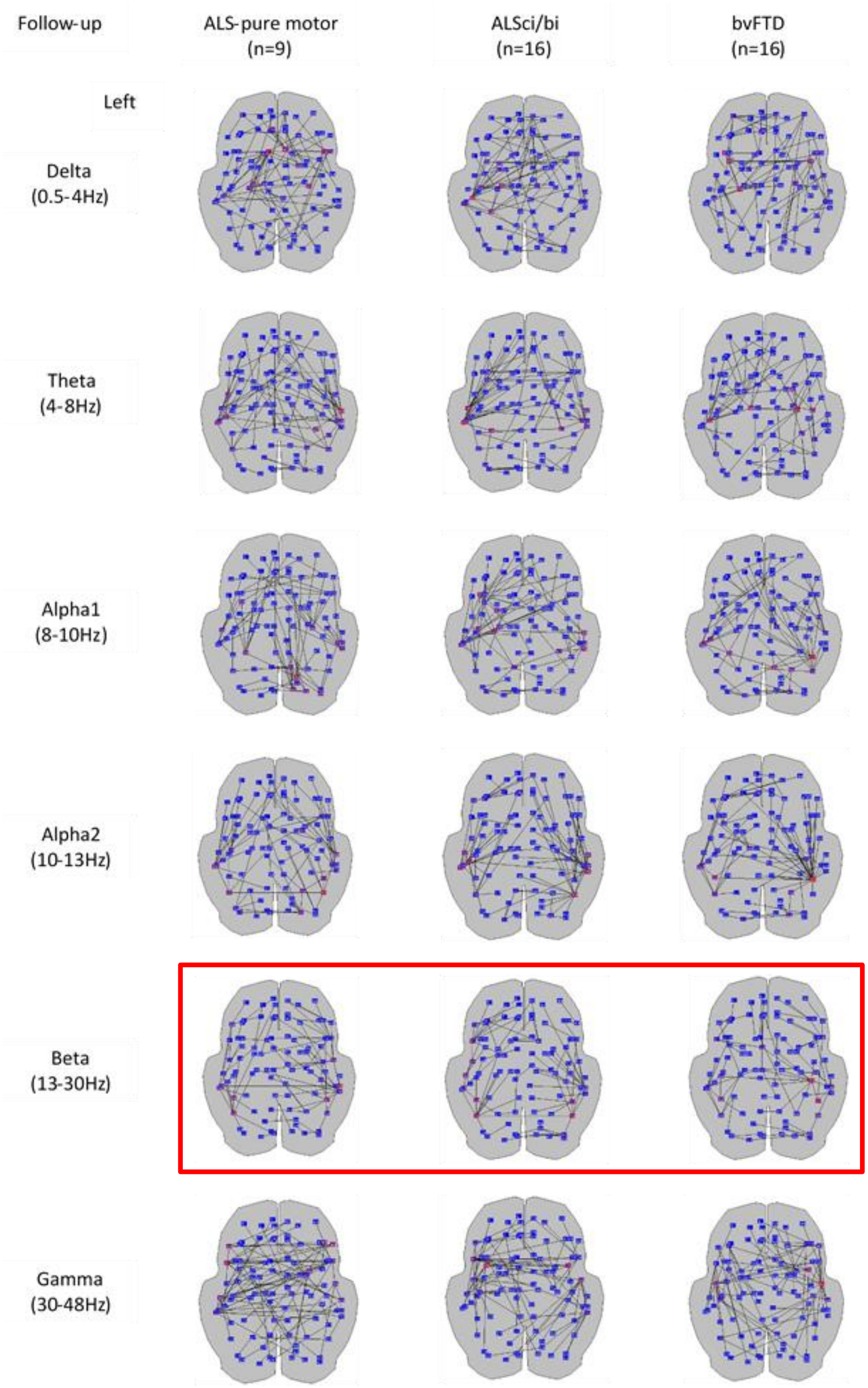

Figure S4. Follow-up MST network changes in ALS-pure motor, ALSci/bi and bvFTD
As in Figure S3, but for the follow-up recordings.

MST – tree hierarchy, leaf fraction, diameter and betweenness centrality

Based on the MST, tree hierarchy, leaf fraction and diameter were calculated to characterise the global topology.[1] Tree hierarchy quantifies the trade-off between large scale integration in the MST and preventing information overload of central nodes, leaf fraction is the fraction of leaf nodes in the MST, where a leaf node is defined as a node with degree one, and diameter is defined as the largest distance (path length) between any two nodes. The betweenness centrality was calculated for each region as a measure of centrality. Betweenness centrality is defined as the fraction of all shortest paths that pass through a particular node.[2-4]

We analysed how MST descriptives (tree hierarchy, leaf fraction and diameter per frequency band) differed between groups. Global MST for the tree hierarchy, leaf fraction and diameter within specific frequency bands were summarized as mean (standard deviation) over subjects and were compared between groups at baseline and follow-up, using the Kruskal-Wallis test. To compare the difference between baseline and follow-up within the different groups the Wilcoxon signed rank test was used.

For each frequency band separately, correction for multiple comparisons was done for the three metrics for the global analysis (tree hierarchy, leaf fraction and diameter) using the false discovery rate (FDR < .05).[5] Betweenness centrality values per ROI were compared between the different groups (at baseline and FU: ALS-pure motor vs ALSci/bi, ALS-pure motor vs bvFTD and bvFTD vs ALSci/bi, and for each group between FU and baseline) by means of permutation analyses, where a null distribution for between-group differences (unpaired t-test) and within groups (baseline – follow-up; paired t-test) was derived by permuting group assignment and calculating a t-statistic after each permutation (total of 10000 permutations), and a correction for multiple comparisons across ROIs was made by using the maximum statistic.[6]

Global MST - Tree hierarchy, Leaf fraction, Diameter

No significant differences between global MST metrics at baseline and follow up were found, nor for within groups comparisons between baseline and follow up (supplemental material Table S2).

MST - Regional betweenness centrality

At baseline, there was a difference between ALS-pure motor and ALSci/bi in the gamma band in the left rolandic operculum, between ALS-pure motor and bvFTD and ALSci/bi and bvFTD in the left anterior cingulate gyrus. Furthermore there was a significant difference between ALSci/bi and bvFTD in the alpha1 band in the right angular gyrus and in the gamma band in the left anterior cingulate gyrus. Although we corrected for multiple testing over ROIs, we did not correct for testing over frequency bands. We therefore refrain from (over)interpretation of these scattered findings.

No significant regional differences between and within groups were found for the other frequency bands.

Table S2. Global MST descriptives for six frequency bands in ALS-pure motor, ALSci/bi and bvFTD

		**ALS-pure motor (n = 9)**		**ALSci/bi (n = 16)**		**BvFTD (n = 16)**	
		BL	FU	BL	FU	BL	FU
Delta	Leaf fraction	.515 (.023)	.521 (.016)	.518 (.018)	.521 (.022)	.530 (.025)	.535 (.034)
	Tree hierarchy	.378 (.015)	.392 (.015)	.377 (.013)	.385 (.018)	.389 (.021)	.391 (.028)
	Diameter	.199 (.010)	.202 (.013)	.198 (.013)	.197 (.011)	.194 (.017)	.192 (.013)
Theta	Leaf fraction	.528 (.016)	.543 (.021)	.535 (.016)	.536 (.016)	.542 (.029)	.543 (.031)
	Tree hierarchy	.388 (.018)	.393 (.015)	.393 (.018)	.388 (.017)	.400 (.017)	.393 (.020)
	Diameter	.190 (.014)	.191 (.018)	.196 (.014)	.191 (.017)	.194 (.018)	.191 (.014)
Alpha1	Leaf fraction	.533 (.021)	.544 (.021)	.526 (.018)	.535 (.016)	.534 (.033)	.540 (.039)
	Tree hierarchy	.403 (.019)	.401 (.009)	.390 (.018)	.393 (.016)	.393 (.020)	.395 (.024)
	Diameter	.194 (.013)	.189 (.010)	.197 (.014)	.194 (.012)	.196 (.017)	.197 (.013)
Alpha2	Leaf fraction	.541 (.022)	.547 (.023)	.535 (.014)	.543 (.021)	.542 (.025)	.545 (.027)
	Tree hierarchy	.401 (.021)	.403 (.018)	.400 (.019)	.399 (.019)	.397 (.015)	.398 (.019)
	Diameter	.195 (.015)	.189 (.012)	.195 (.011)	.192 (.012)	.189 (.012)	.194 (.015)
Beta	Leaf fraction	.570 (.035)	.576 (.036)	.564 (.030)	.569 (.027)	.566 (.033)	.567 (.038)
	Tree hierarchy	.405 (.016)	.421 (.020)	.407 (.018)	.410 (.019)	.410 (.025)	.412 (.030)
	Diameter	.171 (.012)	.178 (.016)	.176 (.020)	.173 (.016)	.184 (.019)	.182 (.017)
Gamma	Leaf fraction	.527 (.028)	.532 (.034)	.521 (.019)	.539 (.035)	.533 (.036)	.536 (.042)
	Tree hierarchy	.378 (.012)	.391 (.021)	.382 (.014)	.390 (.019)	.388 (.016)	.392 (.025)
	Diameter	.194 (.020)	.197 (.022)	.196 (.016)	.189 (.024)	.191 (.029)	.193 (.024)

There were no significant differences between the groups at baseline (BL) or follow up (FU), nor within groups for baseline versus follow up. Amyotrophic lateral sclerosis (ALS) patients – pure motor, ALS with cognitive and/or behavioural impairment including ALS-bvFTD (ALSci/bi), and behavioural variant frontotemporal dementia (bvFTD) patients.

Table S3. ALSci/bi network similarity

	Similarity to MST_{ref}-motor		Similarity to MST_{ref}-bvFTD	
	Baseline	Follow-up	Baseline	Follow-up
Delta	.024 (.015)	.025 (.015)	.038 (.023)	.028 (.022)
Theta	**.045 (.022)**	**.057 (.021)**	.054 (.032)	.048 (.015)
Alpha1	.047 (.020)	.041 (.027)	.041 (.029)	.051 (.023)
Alpha2	.051 (.022)	.056 (.029)	.056 (.028)	.059 (.025)
Beta	.114 (.046)	.086 (.032)	**.116 (.035)**	**.111 (.043)**
Gamma	.029 (.022)	.037 (.019)	.029 (.011)	.046 (.031)

Note: Data are expressed as mean (SD) and based on the mean over epochs (n=8) for every patient. ALS: amyotrophic lateral sclerosis; ci/bi: cognitive and/or behavioural impairment bvFTD: behavioural variant frontotemporal dementia. Bold: significant difference based on the Wilcoxon signed rank test ($p < .05$), FDR corrected.

References

1. Boersma M, Smit DJ, Boomsma DI, De Geus EJ, Delemarre-van de Waal HA, Stam CJ. Growing trees in child brains: graph theoretical analysis of electroencephalography-derived minimum spanning tree in 5- and 7-year-old children reflects brain maturation. Brain Connect 2013;3:50-60.
2. van Dellen E, Sommer IE, Bohlken MM, et al. Minimum spanning tree analysis of the human connectome. Hum Brain Mapp 2018;39:2455-2471.
3. Tewarie P, Hillebrand A, Schoonheim MM, et al. Functional brain network analysis using minimum spanning trees in Multiple Sclerosis: An MEG source-space study. NeuroImage 2014;88:308-318.
4. Wang H, Hernandez JM, Van Mieghem P. Betweenness centrality in a weighted network. Physical Review E 2008;77:046105.
5. Benjamini Y, Hochberg Y. Controlling The False Discovery Rate - A Practical And Powerful Approach To Multiple Testing. J Royal Statist Soc, Series B 1995;57:289-300.
6. Nichols TE, Holmes AP. Nonparametric permutation tests for functional neuroimaging: a primer with examples. Human brain mapping 2002;15:1-25.
